# Tetraploidy Enhances Boron-Excess Tolerance in Carrizo Citrange (*Citrus sinensis* L. Osb. × *Poncirus trifoliata* L. Raf.)

**DOI:** 10.3389/fpls.2016.00701

**Published:** 2016-05-25

**Authors:** Marta Ruiz, Ana Quiñones, Belén Martínez-Alcántara, Pablo Aleza, Raphaël Morillon, Luis Navarro, Eduardo Primo-Millo, Mary-Rus Martínez-Cuenca

**Affiliations:** ^1^Centro de Protección Vegetal y Biotecnología, Instituto Valenciano de Investigaciones AgrariasMoncada, Spain; ^2^Centro de Citricultura y Producción Vegetal, Instituto Valenciano de Investigaciones AgrariasMoncada, Spain; ^3^UMR AGAP, Centre de Coopération Internationale en Recherche Agronomique Pour le DéveloppementMontpellier, France

**Keywords:** 4x, citrus, rootstock, poliploidy, Casparian strip, NIP5, BOR1

## Abstract

Tetraploidy modifies root anatomy which may lead to differentiated capacity to uptake and transport mineral elements. This work provides insights into physiological and molecular characters involved in boron (B) toxicity responses in diploid (2x) and tetraploid (4x) plants of Carrizo citrange (*Citrus sinensis* L. Osb. × *Poncirus trifoliata* L. Raf.), a widely used citrus rootstock. With B excess, 2x plants accumulated more B in leaves than 4x plants, which accounted for their higher B uptake and root-to-shoot transport rates. Ploidy did not modify the expression of membrane transporters NIP5 and BOR1 in roots. The cellular allocation of B excess differed between ploidy levels in the soluble fraction, which was lower in 4x leaves, while cell wall-linked B was similar in 2x and 4x genotypes. This correlates with the increased damage and stunted growth recorded in the 2x plants. The 4x roots were found to have fewer root tips, shorter specific root length, longer diameter, thicker exodermis and earlier tissue maturation in root tips, where the Casparian strip was detected at a shorter distance from the root apex than in the 2x roots. The results presented herein suggest that the root anatomical characters of the 4x plants play a key role in their lower B uptake capacity and root-to-shoot transport.

**Highlights:**

Tetraploidy enhances B excess tolerance in citrange Carrizo
Expression of NIP5 and BOR1 transporters and cell wall-bounded B are similar between ploidies
B tolerance is attributed to root anatomical modifications induced by genome duplication
The rootstock 4x citrange carrizo may prevent citrus trees from B excess.

## Introduction

Boron (B) is an essential micronutrient required in major physiological functions for normal higher plant growth and development (Brown et al., 2002), as it participates in cell wall structure formation through the borate-diol bonding of two rhamnogalacturonan II molecules (O‘Neill et al., 2004; Funakawa and Miwa, 2015). It is also involved in root elongation, carbohydrate metabolism, phenol accumulation, pollen-tube growth, and cell membrane integrity (Brown et al., 2002; Herrera-Rodríguez et al., 2010).

Citrus is a sensitive crop to excess B, whose toxicity causes major disorders that lead to reduced tree vigor and loss of yield. B toxicity symptoms usually appear on older leaves, first as leaf tip and margin yellowing or mottling, then with a brownish burnt appearance, and finally end in premature fall (Chapman, 1968; Papadakis et al., 2004; Grattan et al., 2015; Martinez-Cuenca et al., 2015). Several recent related studies, carried out in the B-tolerant specie *Citrus sinensis* (L.) Osb. and the B-sensitive *C. grandis* (L.) Osb., have demonstrated that these phenotypes correlate with diferential changes in leaf proteome, specifically in those proteins related to photosynthesis and detoxification (Sang et al., 2015), gene expression (Guo et al., 2014) and plant anatomy (Huang et al., 2014). The microRNA miR397 has been identified as a regulator element, playing a pivotal role in tolerance to B-toxicity by targeting two laccase genes, *LAC17* and *LAC4*, which are responsible for secondary cell wall synthesis in response to B-toxicity. Cell wall deposits may limit the inflow of B transported via xylem to the leaf mesophyll (Huang et al., 2016). In trifoliate orange (*Poncirus trifoliata* L. Raf.), excess B reduced the expression of miR397 and increased the expression of its target *LAC7* (Jin et al., 2016). In other woody species such as *Malus* × *domestica* Borkh., B excess has been described to inhibit pollen germination and tube development (Fang et al., 2016).

Boron in soil solution is present mainly as BA or borate, but B uptake by roots is performed mainly as the former (Brown et al., 2002). Boron toxicity occurs mostly by irrigation with high B concentrations in water, or in arid and semiarid areas where water reaches the topsoil by capillarity and then evaporates to cause B accumulation (Nable et al., 1997). This is the case of the Segura river lower basin in Spain, a semi-arid region where drought, water deficit and water quality are common factors that limit agricultural production. Increasing demand has rendered the use of reclaimed water for irrigation necessary. Most treated wastewaters in this area have a high enough B concentration to harm citrus (Grattan et al., 2015). The range between critical soil B deficiency and excess levels is narrow, so regulating its absorption and transport in plants is essential to maintain the organism’s general B homeostasis. At high soil B levels, B absorbed by roots accumulates in leaves as they age to the extent that toxicity can occur. Inside plants, B is bound mostly to cell wall structures (insoluble B pool) or concentrates in apoplastic fluids (soluble B pool). A high amount of B bounded to cell wall has been related to B tolerance in *C. sinensis*, while abundant soluble B pool was correlated to the sensitive specie *C. grandis* (Huang et al., 2014). The soluble portion that enters cells is very low anyway (Liu et al., 2013), and vacuolar compartmentation might also play a key role in cell B tolerance (Martinez-Cuenca et al., 2015).

Roots absorb water and solutes through their surface, and flux can reach the xylem vessels and be transported to shoots via apoplast, passing by intercellular spaces or via symplast across the cytoplasms (Brown et al., 2002; Takano et al., 2008). The non-specific apoplastic transport pathway is regulated by two suberin barriers located at the exodermis and the endodermis (Peterson et al., 1993; Baxter et al., 2009). The former limits the entry of water and solutes through the root surface (Hose et al., 2001) and its suberisation patterns control these processes along root length (Gambetta et al., 2013). The latter restricts the apoplastic flow into the xylem, and prevents uncontrolled leakage of water and mineral elements (Chen et al., 2011). Then, nutrients such as B, need to pass the Casparian strip to be transported from the root cortex to the xylem, which occurs in two steps: (1) uptake into endodermal cells, and (2) export out to the stele (Takano et al., 2008; Miwa and Fujiwara, 2010). Furthermore, the symplastic pathway includes three transmembrane transport events: (1) entry into epidermal, cortical, or endodermal cells; (2) cell-to-cell displacement through plasmodesmata; and, (3) xylem loading from pericycle or xylem parenchyma cells (Takano et al., 2008).

It has been believed that the passive diffusion of BA across the plasma membrane constitutes the major mechanism of B uptake by cells (Takano et al., 2008; Miwa and Fujiwara, 2010) based on the high permeability of lipid bilayers to undissociated BA (Dordas et al., 2000). Thus, B uptake by plants should be the result of a non-metabolic process determined by the external concentration, membrane permeability, formation of B complexes in roots, translocation of B within the plant and E (Raven, 1980). However, evidence indicates that under restricted B supply conditions, passive B influx seems inadequate for plants to fulfill B demands (Pfeffer et al., 1999), from which B uptake and transport may be facilitated by a metabollically active and inducible carrier-mediated transport process (Brown et al., 2002; Takano et al., 2008; Miwa and Fujiwara, 2010).

At low external levels, B acquisition needs B transport proteins (BORs) and NIPs to facilitate translocation from roots to shoots and absorption from soil, respectively. The first B transporter identified in *Arabidopsis thaliana* (L.) Heynh was AtBOR1, an essential efflux-type transporter for xylem loading of B in roots under B limitation (Takano et al., 2002), which is localized mainly in the plasma membrane of root pericycle cells and whose overexpression enhances root-to-shoot B translocation under restricted B conditions (Miwa et al., 2006). The removal of BOR1 protein from membranes in response to high B levels may occur by endocytosis and degradation in vacuoles (Takano et al., 2005, 2010), as a mechanism involved in the regulation of B transport from roots to shoots at high B levels. *CmBOR1* has been recently characterized as a B transporter in *C. macrophylla* Wester (Cañon et al., 2013).

Another membrane protein that facilitates B influx to root cells is *NIP5;1* (Takano et al., 2006a), a BA channel member of the major intrinsic protein family that mediates the passive flow of water and small uncharged molecules. It is localized in the plasma membrane on the outer side of epidermal cells in roots (Takano et al., 2006b, 2010), where it is required for B uptake from the root surface. *NIP5;1* activity is up-regulated in response to B deprivation (Takano et al., 2006a), and the overexpression of *AtNIP5;1* improves short-term B uptake under B-deficient conditions and enhances root elongation (Kato et al., 2009). Recently, Tanaka et al. (2011) suggested that B-dependent degradation of *NIP5;1* mRNA is important for plants to acclimatize to high B conditions. *CiNIP5* has also been characterized in CC (*C. sinensis* × *P. trifoliata*) (An et al., 2012).

Doubled diploid (4x) plants are clones of their 2x ancestors, but differ from them in a doubled number of somatic chromosomes. In apomictic 2x genotypes of citrus, 4x usually arise spontaneously by chromosome set doubling in maternal nucellar cells, which then form somatic embryos (Barrett and Hutchison, 1978; Aleza et al., 2011). It has been shown by molecular markers analyses that 2x and their derived 4x genotypes are genetically identical (Aleza et al., 2011).

In citrus, some phenotypic differences between ploidy levels have been described (Cameron and Frost, 1968; Barrett and Hutchison, 1978; Romero-Aranda et al., 1997; Allario et al., 2011). When compared with 2x plants, 4x present thicker greener leaves and shorter thicker roots. Shorter diameters and higher SRL influence roots to have greater radial hydraulic conductivities (*Lp*_r_) which comprises radial path for water and solute movement from the root surface to the xylem. Differences in *Lp*_r_ among distinct citrus rootstocks have also been linked to anatomical characteristics, such as suberisation and exodermis thickness (Huang and Eissenstat, 2000). When soil moisture is available, *Lp*_r_ represents the major limiting factor for water and solute uptake. The differences in this parameter between rootstocks can result in restricted transport to shoots, which influences the leaf water status and, in turn, plant growth and physiological responses. Accordingly, the shoot growth rate of several citrus rootstock seedlings depends on *Lp*_r_ to a great extent (Syvertsen and Graham, 1985). As SRL, and therefore root *Lp*_r_, is lower in 4x trees than in 2x ones, growth of 4x is slower compared to 2x, and results in trees with a compact structure (Barrett and Hutchison, 1978). For the same reason, *E* is also lower in 4x plants than in 2x ones (Syvertsen et al., 2000), as with leaf stomatal conductance (Allario et al., 2011). Moreover, the thicker mesophyll of the 4x citrus plants may result in increased internal diffusive resistances, which lower the *A*co_2_ in comparison to 2x plants (Romero-Aranda et al., 1997). Finally, differences in the stomata size and density beween 2x and 4x (Allario et al., 2011) may lead to large changes of estomatal conductance and *E* (Franks and Farquhar, 2007). All these factors may be operative for determining the smaller size characteristic of 4x trees.

Some 4x citrus could be used as rootstocks since the physiology of citrus trees is deeply influenced by root characteristics, which play a key role in the capacity to uptake and transport mineral elements. In this way, citrus rootstocks differ in terms of their ability to exclude toxic compounds from being accumulated in leaves; consequently, the use of tolerant rootstocks is considered a suitable system to agronomically manage toxicity problems given the excess of some elements (Walker et al., 1984). Moreover, a recently published research (Mesquita et al., 2016) highlights the key role of citrus rootstocks for B management in citrus orchards.

In this work we studied the behavior of 2x and 4x plants of CC, which were tested as seedlings or grafted rootstocks with high B levels in the external medium. The ultimate purpose was to provide insights into the morphological, anatomical, physiological, and molecular characters linked to tetraploidy that determine differences in tolerance to B toxicity. This hybrid was chosen because it is the most widespread rootstock in the Spanish citrus industry, where it is currently established in approximately 75% of orchards, and is widely used in other citrus areas. Moreover, citranges are considered moderately sensitive to B toxicity (Grattan et al., 2015).

## Materials and Methods

### Plant Material

The plant material used for this study was originated from 2x and 4x CC trees of the Citrus Germplasm Bank of pathogen-free plants at the Instituto Valenciano de Investigaciones Agrarias (IVIA). The 4x genotype was selected in 1994 among young seedlings from the 2x genotype. Previous routine analysis by SSR markers indicated that both genotypes were genetically identical.

### Methods

#### Experimental Conditions

##### Experiment 1

The seeds of 2x and 4x CC were sterilized for 5 min in a 2% v/v commercial bleach solution (0.5 M NaClO) prior to seed coat removal, rinsed three times with sterilized deionised water, and transferred to media that contained distilled water with 0.4% agar (Difco Bacto) and pH was adjusted to 6.0. Media were previously autoclaved at 120°C for 20 min and distributed in 150 × 25 mm tubes (40 mL per tube). Seeds (one seed per tube) were germinated in a growth chamber (Sanyo MCR-350H, Sanyo Electric Biochemical Co.) at 20-22/26–28°C night/day temperatures, 80% relative humidity (RH) and 250 μmol m^-2^ s^-1^ photosynthetic photon flux density, 16 h day^-1^.

After 20 days, seedlings were selected for uniformity and transferred individually after removing cotyledons to 150 × 25 mm plastic tubes (50 mL per tube) that contained basic nutrient solution [5 mM Ca(NO_3_)_2_, 1.5 mM KNO_3_, 1 mM MgSO_4_, 1.2 mM H_3_PO_4_, 20 μM Fe-EDDHA, 7.65 μM ZnSO_4_⋅7H_2_O, 54.4 μM MnSO_4_⋅H_2_O, 0.5 μM CuSO_4_⋅5H_2_O] to which either 50 or 400 μM H_3_BO_3_ were added (Ct or +B, respectively). All the media were supplemented with 0.25% agar, adjusted to pH 6.0 and sterilized, as previously described, before transplanting. Seedlings were kept for 45 days in the same growth chamber under the above conditions. At the beginning of the assay (initial plant growth) and after 45 days of B treatment, seedlings were removed from culture tubes, rinsed with distilled water, and divided into leaves, stems and roots. These organs were fresh-weighed and the length of stems and roots was measured. Fresh samples of organs were taken for the analytical and molecular determinations, and the remaining parts were reweighed after being dried in a forced draft oven at 70°C for 48 h to obtain the DW/FW ratio.

##### Experiment 2

Carrizo citrange 2x and 4x seeds were germinated in a glasshouse using a sterile substrate composed of peat, coconut fiber, sand and perlite (50:25:20:5), supplemented with 1.38 g kg^-1^ calcium superphosphate and irrigated twice weekly with the following basal nutrient solution at half strength: 5 mM Ca(NO_3_)_2_, 1.4 mM KNO_3_, 2 mM MgSO_4_, 0.6 mM H_3_PO_4_, 20 μM Fe-EDDHA, 7.6 μM ZnSO_4_⋅7H_2_O, 0.50 μM CuSO_4_⋅5H_2_O, 50 μM H_3_BO_3_, 0.50 μM MoO_3,_ and 54 μM MnSO_4_⋅H_2_O. The nutrient solution pH was adjusted to 6.0 with 1 M KOH or 1 M H_2_SO_4_. After 8 weeks, seedlings were selected according to uniformity of size and were transplanted individually to opaque plastic 0.5 L pots filled with a substrate composed of peat, coconut fiber, sand and perlite (40:25:25:10). The seedlings of each ploidy (2x and 4x) were then separated into four groups and fed with the above described basal nutrient solution, which contained 50, 200, 400, or 800 μM H_3_BO_3_. Plants were severely pruned to force new stems and leaves to develop under the B treatment. Immediately after this practice, six plants of each ploidy were separated and carefully removed from pots. Then roots were washed with tap water to eliminate the substrate and pruned plants were fresh-weighed individually to record weights at the beginning of treatments. Twelve other seedlings per ploidy and treatment were randomized over the experimental area, and a row of plants, not included in the experiment, was placed around the perimeter as a border row.

Seedlings were grown under glasshouse conditions with supplementary light (250 μmol m^-2^ s^-1^, 400–700 nm) to extend the photoperiod to 16 h. The temperature ranges were 16–18°C at night and 26–28°C during the day. RH was maintained at approximately 80%. Pots were irrigated twice weekly using 300 mL of solution per pot in each watering event. Excess solution was drained from the pot to avoid salt accumulation in the substrate. Six plants per genotype and treatment were maintained for 5 weeks under these conditions and samples for the B analysis (two mid-stem leaves) were taken weekly from each plant. Seedlings were carefully removed from pots and roots were washed with tap water to eliminate the substrate. Whole seedlings were rinsed with deionised water. Leaves, stems and roots were separated and weighed individually. Roots were dissected into taproots and lateral roots to visually count the total root tip number per plant.

Growth and leaf damage was quantified in six other seedlings from genotypes 2x and 4x, subjected to 50 and 800 μM H_3_BO_3_ under the above conditions for 10 weeks. For this purpose, the total leaf area was measured with an LI-300 area-meter (LI-COR). Then the burnt leaf area was cut and removed from the leaf. The remaining healthy area was measured as described above. Leaf injury was expressed as the ratio between the damaged leaf area and the total leaf area (as a %). Total and abscised leaves per plant were counted and the percentage of defoliation was calculated. All the organs were dried in a forced draft oven at 70°C for 48 h until constant DW for growth measurement.

##### Experiment 3

This experiment was conducted in an experimental orchard located in Elche (Alicante, Spain) (38°14′53.57″N; 0°41′46.9″W), a semi-arid region in the Segura river lower basin. The study site has an annual average temperature of 17.9°C, annual average rainfall of 290.2 mm, and annual average relative air humidity of 65%. The soil texture within the first 50 cm depth was classified as clay loam soil (27% sand, 38% clay, and 35% silt, USDA). The main chemical soil characteristics were: 8.5 pH, 32.7% total calcium carbonate (w/w), 1.63% organic matter (w/w), and 0.56 mg kg^-1^ B concentration in soil. The B concentration in irrigation water was variable. The main irrigation source was reclaimed water (0.7 mg B L^-1^) but good quality water was applied from Tajo-Segura water transfer canal (0.05 mg B L^-1^) when avaible. In 2009 the orchard was planted with VL orange (*C. sinensis*) trees grafted onto rootstocks 2x and 4x CC. Tree planting distances were 5 × 5 m. A randomized block design with three blocks that contained two experimental plots per block (one per rootstock ploidy) was used in this assay. The standard plot was made up of four trees that formed a square. An extra row of guard trees separated one experimental block from another.

All the plants were cultivated in accordance with local management practices, including irrigation, fertilization, pruning, and pest control. The leaf B concentration was determined in spring flush leaves from the non-fruiting shoots collected in November of 2012, 2013, and 2014. For the B analysis, six leaves per tree were randomly sampled from the canopy periphery. Leaf samples were pooled from the four trees of each plot, which was analyzed independently.

Tree height and diameter were measured in post-planting year 6 (2015). From these data, canopy volume (V) was calculated according to Turrell’s equation:

$$V(m^{3})=0.5236\times H\times D^{2}$$

where H is the height of the tree and D is the maximum canopy diameter.

Fruits were harvested upon ripening and the yield of each tree was weighed individually. Yield efficiency (kg m^-3^) was calculated as the weight of production (kg tree^-1^)/canopy volume (m^3^ tree^-1^).

### Analytical Methods

#### Ploidy Level Determination

Carrizo citrange plants, used as seedlings or rootstocks in the different experiments, were obtained from the seeds of the above-mentioned 2x and 4x CC trees, and the ploidy of nucellar seedlings generated (which had to be genetically identical to the mother plant) was checked.

The ploidy level was determined by flow cytometry. Samples consisted in a small piece (∼0.5 mm^2^) of leaf taken from all the plants used in the experiments. Samples were chopped with a razor blade in the presence of nuclei isolation solution (High Resolution DNA Kit Type P, solution A; Partec). Nuclei were filtered through a 30-μm nylon filter and stained with DAPI (4′,6-diamine-2-phenylindol) solution (High-Resolution DNA Kit Type P, solution B; Partec). After 5 min of incubation, stained samples were run in a Ploidy Analyzer (PA; Partec) flow cytometer, equipped with an HBO 100-W high-pressure mercury bulb, and with both KG1 and BG38 filter sets. Histograms were analyzed by the dpac software, v2.0 (Partec), which determines peak position, coefficient of variation, and the relative ploidy index of samples.

#### RNA Extraction and qRT-PCR Analysis

The relative expression of genes *NIP5* and *BOR1*, measured by a qRT-PCR analysis, was evaluated in the roots of the plants grown *in vitro*. Fresh samples (0.1 g) were ground in a mortar in liquid N_2_. The total plant RNA from leaf and root tissues was extracted with the RNeasy Plant Mini Kit (Qiagen) and treated with DNase I (*Ambion, Thermo Fisher Scientific Inc.*), following the manufacturers’ instructions. RNA quality and concentration were assessed in an ND-1000 full spectrum UV-Vis spectrophotometer (Nanodrop Technologies, Thermo Fisher Scientific). Then 2 μg of DNase-treated RNA was reverse-transcribed with oligo (dT) 23 primer in a 20 μl reaction mixture using RevertAid M-MuLV reverse transcriptase (*RevertAid, Thermo Fisher Scientific Inc*.). After heat inactivation, 1 mL of the threefold diluted cDNA was used as a template to perform qPCR in a total volume of 20 μL using Perfecta SYBR Green FastMix (Quanta Bioscience). Reactions were run in a LightCycler 480 system (*Roche Applied Science*) under the following conditions: 95°C for 3 min and 40 cycles of PCR (95°C for 15 s and 60°C for 1 min). The primer pairs used for each gene quantification were those described by previous authors (An et al., 2012; Cañon et al., 2013) and are shown in **Table 1**. Three independent biological replicates with three technical replicates were performed. Data were normalized against the clementine mandarin (*C. clementina Hort. ex Tan.)* putative actin gene (**Table 1**).

**Table 1:** List of the primers used for quantitative reverse transcription PCR.

Annotation	Code	Forward/Reverse primer	Reference
*NIP5*	JQ277272^1^	5′-AGCTTCCCGGATATTCCAGT-3′	An et al., 2012
		5′-ACTATGGGTCCTGCTGTTGC-3′	
*BOR1*	EF581174^1^	5′-GGGCATATAGTCCCCGTGTT-3′	Cañon et al., 2013
		5′-CCGGGACTGGGAACTTTC-3′	
*ACT*	clementine0.9_013110m^2^	5′-CAGTGTTTGGATTGGAGGATCA-3′	Agüero et al., 2014
		5′-TCGCCCTTTGAGATCCACAT-3′	


*^1^Accession number in NCBI reference sequence database (http://www.ncbi.nlm.nih.gov).*

*^2^Code refers to the transcript name in the database available in the International Citrus Genome Consortium (http://www.phytozome.net/search.php).*

#### Total B Concentration

Samples of organs were fresh-weighed, washed in a 0.01% Tween-20 (*Sigma-Aldrich Corp*.) v/v solution, gently rinsed with distilled water and oven-dried at 70°C for 48 h until constant DW. The total B (B_T_) concentration was measured in dry tissues (0.5 g), which were burnt in a muffle furnace at 550°C for 12 h and then extracted with 0.1 N HCl (Hiperpur Panreac) to a final volume of 5 mL. The B_T_ concentration was determined by inductively coupled plasma atomic emission spectroscopy (ICP-AES iCAP 6000, Thermo Scientific).

#### B Partitioning

The B concentration in the soluble and cell wall fractions was measured in the leaves and roots from *in vitro* cultured seedlings (Experiment 1). Samples were frozen and ground in a mortar into fine powder in liquid N_2_. Powder (0.5 g) was homogenized with 10 volumes of ice-cold water and centrifuged at 1000 rpm for 10 min. The precipitate was washed with 10 volumes of ice–cold water and recentrifuged. The residue was washed three times with 10 volumes of 80% ethanol, once with 10 volumes of the methanol: chloroform mixture (1:1, v/v), and once with 10 volumes of acetone. The combined supernatants were defined as the soluble B fraction. The insoluble pellet was used for the B bound to cell wall. All the fractions were dried, extracted and measured similarly to the B_T_ samples.

#### B Uptake and Transport Rate Calculations

The net uptake rate (μmol g^-1^ root DW day^-1^) was calculated from the total B accumulated in seedlings by Pitman’s equation:

$${Uptake}_{rate}=\left[\frac{In{\;R}_{45}-In{\;R}_{0}}{R_{45}-R_{0}}\right]\times \left[\frac{C_{T45}-C_{T0}}{t_{45}-t_{0}}\right]$$

where *R* is the root dry mass and *C*_T_ is the total element content of the whole plant at the beginning (*t*_0_) and end (*t*_45_) of the assay.

The net B transport rate (μmol g^-1^ root DW day^-1^), which quantifies root-to-shoot B translocation, was calculated as follows:

$${Transport}_{rate}=\left[\frac{In{\;R}_{45}-In{\;R}_{0}}{R_{45}-R_{0}}\right]\times \left[\frac{C_{A45}-C_{A0}}{t_{45}-t_{0}}\right]$$

where R is the root dry mass and *C*_A_ is the content of the element in the aerial part at the beginning (*t*_0_) and end (*t*_45_) of the assay.

### Light Microscopy

Ten days after being transferred to the tubes that contained culture media, the 2x and 4x CC seedlings that grew *in vitro* (as in Experiment 1), which showed a unique straight root, were removed from the tubes and were carefully washed with distilled water.

#### Sectioning

Roots were separated under water using a razor blade and then, with the help of a Leica MZ8 stereomicroscope, they were free hand-sectioned as thinly as possible at different distances from the root apex on a wet surface. Sections were transferred from the blade to a drop of water on a microscope slide. The water from the drop was then partially absorbed by tissue paper before applying clearing solutions.

#### Preparation of Solutions

Clearing solution: Lactic acid (85%, Sigma) saturated with chloral hydrate (crystallized >98%, Sigma).

Staining solutions:

(a) Berberine hemisulfate (Sigma) dissolved at 0.1% (w/v) in lactic acid, 85%.
(b) Aniline blue (Merck) dissolved at 0.5% (w/v) in distilled water.

#### Clearing and Staining Procedures

Sections were cleared and stained following the procedure described by Lux et al. (2005), to detect suberin and lignin in plants by fluorescent staining, which improves previous methods for viewing Casparian strip in root exodermal and endodermal cells.

The root sections that floated in the drops of clearing solution on microscope slides were heated over a water bath in covered Petri dishes for 1 h at 70°C. The clearing solution was absorbed with tissue paper and sections were thoroughly washed with distilled water, added several times and absorbed with tissue paper.

Root preparations were stained with solution *a*, which was applied as droplets on the slide and heated over a water bath in covered Petri dishes at 70°C for 30 min. Samples were washed carefully with distilled water, which was then absorbed by tissue paper. Post-staining was performed with solution *b*, applied to the slide for 5 min at room temperature. After rinsing the stain, sections were mounted in water to be observed.

#### Microscopy and Photography

Root sections were observed under an Eclipse e600 fluorescence microscope (Nikon) with ultraviolet illumination (UV-2A: ex 330–380 filter). Images were captured by a DS-Ri1 camera (Nikon) and the accompanying software.

### Statistical Analysis

Data were subjected to analysis of variance (ANOVA). Means were separated using Duncan’s multiple range test at *P* < 0.05 with the Statgraphics Plus, version 5.1 (Statistical Graphics, Englewood Cliffs) software. The interaction between multiple main effects (ME) was only considered if the involved ME were independently significant, otherwise it was indicated as not considered (nc).

## Results

### Boron Excess Effect on 2x and 4x *In Vitro* Seedlings

#### Differences Found in Plant Biomass and Size

In the seedlings cultured *in vitro* under normal B conditions (Ct), the DW of the 4x leaves, stems and roots was higher (2.0-, 2.1- and 1.3-fold, respectively) than in the 2x ones (**Table 2**). Consequently, the whole plant DW of the 4x seedlings was approximately 1.8-fold higher than in the 2x ones. This effect was linked to the bigger sized cotyledons of the 4x seeds in comparison to the 2x cotyledons, which increases the reserve availability for early 4x seedling growth.

**Table 2:** Dry weight (mg) of organs and whole plants, stem and root length (cm) and SRL (m g^-1^) of the 2x and 4x CC seedlings grown *in vitro* (Experiment 1) for 45 days in nutrient media supplemented with 50 μM (Ct) or 400 μM (+B) H_3_BO_3_.

	2x		4x		ANOVA	
			
	Ct	+B	Ct	+B	P	T
**Dry weight (mg)**						
Leaf	15.9^b^	12.6^b^	32.9^a^	36.5^a^	^∗∗∗^	ns
Stem	6.1^b^	5.5^b^	12.8^a^	15.7^a^	^∗∗∗^	ns
Root	13.7^b^	11.2^b^	17.8^a^	21.5^a^	^∗∗^	ns
Whole plant	35.7^b^	29.3^b^	63.4^a^	73.7^a^	^∗∗∗^	ns
**Length (cm)**						
Stem	2.8^b^	2.6^b^	4.7^a^	5.3^a^	^∗∗∗^	ns
Root	18.4	14.9	15.5	15.3	ns	ns
SRL (m g^-1^)	13.5^a^	13.5^a^	8.8^b^	7.4^b^	^∗∗∗^	ns


*Values are means of six plants (*n* = 6). Ploidy (P) and treatment (T) effects tested by two-way ANOVA are indicated as follows: ^∗∗^*P* < 0.01; ^∗∗∗^*P* < 0.001; ns, not significant. Different letters in rows indicate significant differences at *P* < 0.05 using Duncan’s multiple range test.*

B excess (+B) did not significantly reduce the organ DW of the 2x and 4x seedlings compared with their respective controls (Ct). Stems were 40.5% shorter in the 2xCt seedlings than in the 4xCt ones, and length in both cases was not affected by B treatments. Root length was similar in the 2x and 4x seedlings, irrespectively, of the external B level. Thus SRL were more than 50% longer in the 2x plants than the 4x ones for both B-treatments (**Table 2**). These data indicate that the 4x roots were thicker than the 2x ones, as shown in **Table 3**, which presents the values of several root parameters measured at the beginning of the experiment. The root cross-sectional diameter and area, the root cortex area and the root stele diameter and area measured at 20 mm from root apex were, respectively, 44.1, 107.1, 104.0, 50.5, and 126.7% bigger in the 4x roots than in the 2x ones.

**Table 3:** Root cross section parameters of the 2x and 4x CC seedlings cultured *in vitro* measured at 20 mm from root apex.

	2x	4x	ANOVA
Cross section diameter (μm)	593.5^b^	855.3^a^	^∗∗∗^
Cross section area (mm^2^)	0.28^b^	0.58^a^	^∗∗^
Cortex area (mm^2^)	0.25^b^	0.51^a^	^∗∗∗^
Stele diameter (μm)	195.2^b^	293.8^a^	^∗∗∗^
Stele area (mm^2^)	0.03^b^	0.07^a^	^∗∗∗^


*Values are means of six plants (*n* = 6). Significant differences for ploidy (P) tested by ANOVA are indicated as follows: ^∗∗^*P* < 0.01; ^∗∗∗^*P* < 0.001. Different letters in rows indicate significant differences at *P* < 0.05 using Duncan’s multiple range test.*

#### Boron Accumulation in Plants

**FIGURE 1:**
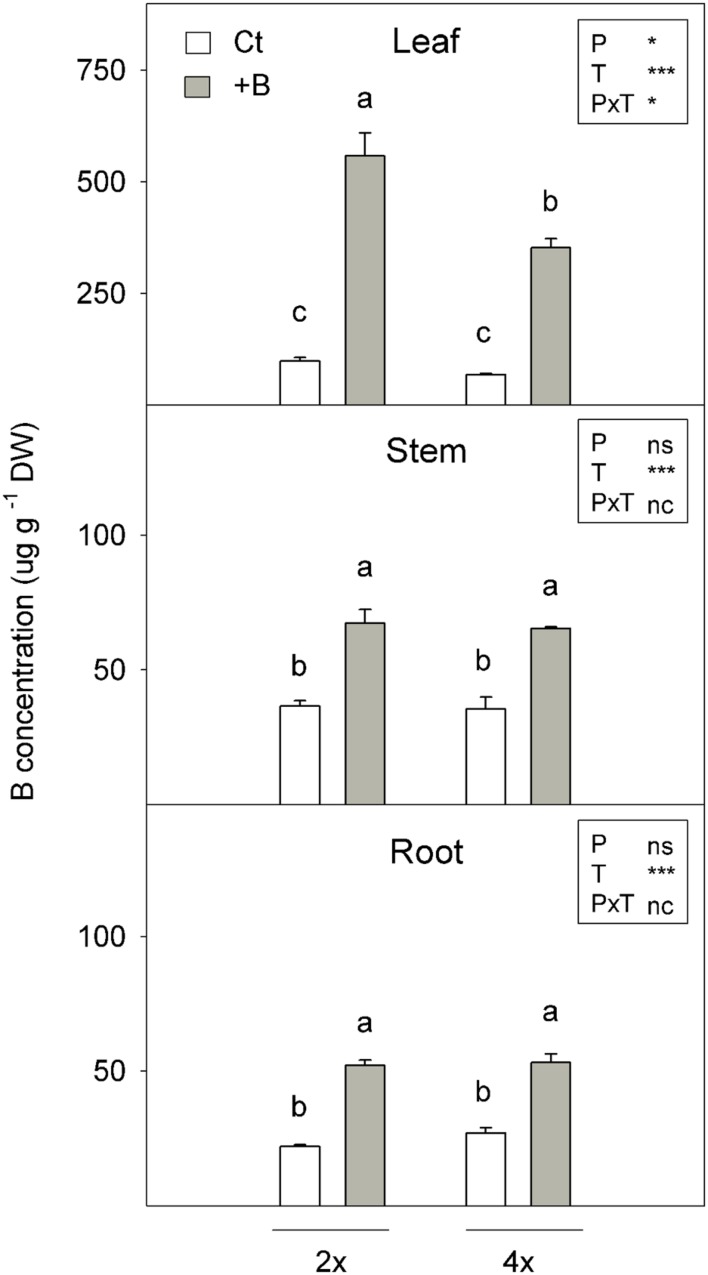
**Boron (B) concentration (μg g^-1^ DW) measured in the 2x and 4x CC seedlings grown *in vitro* (Experiment 1) for 45 days in nutrient media supplemented with 50 μM (Ct) or 400 μM (+B) H_3_BO_3_.** Values are the means ± SE of six plants (*n* = 6). Ploidy (P) and treatment (T) effects tested by two-way ANOVA are indicated as follows: ^∗^*P* < 0.05; ^∗∗∗^*P* < 0.001; ns, not significant; nc, not considered. Different letters indicate significant differences at *P* < 0.05 using Duncan’s multiple range test.

The seedlings grown *in vitro* at 400 μM H_3_BO_3_ (+B) for 45 days had higher B concentrations in leaves, stems and roots than those subjected to 50 μM B (Ct) (**Figure 1**). Tetraploids that underwent +B treatments only differed from diploids in leaf B concentration terms, while stem and root concentrations were similar in both genotypes. The 2x+B leaves accumulated 58% more B than the 4x+B ones (**Figure 1**).

#### Diferences in B uptake and Transport Rates between 2x and 4x Plants

**FIGURE 2:**
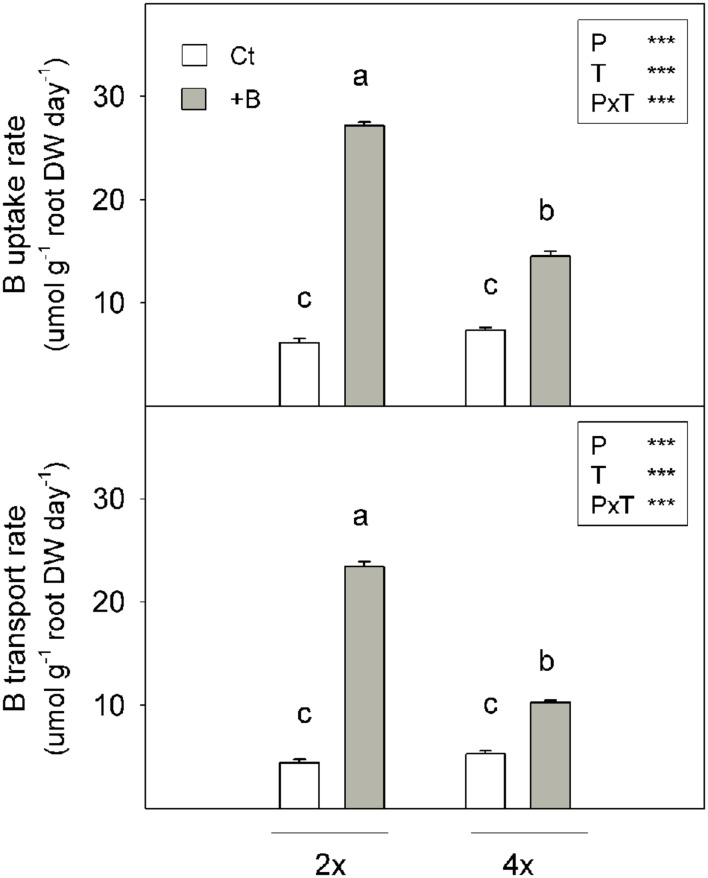
**Boron (B) uptake rate (μmol g^-1^ root DW day^-1^) and B transport rate (μmol g^-1^ root DW day^-1^) measured in the 2x and 4x CC seedlings grown *in vitro* (Experiment 1) for 45 days in nutrient media supplemented with 50 μM (Ct) or 400 μM (+B) H_3_BO_3_.** Values are the means ± SE of six plants (*n* = 6). Ploidy (P) and treatment (T) effects tested by two-way ANOVA are indicated as follows: ^∗∗∗^*P* < 0.001. Different letters indicate significant differences at *P* < 0.05 using Duncan’s multiple range test.

Diploid and 4x plants subjected to Ct treatment had similar B uptake and root-to-shoot transport rates (**Figure 2**). These parameters increased under the +B treatment, irrespectively, of ploidy. Nevertheless, the 2x+B seedlings displayed a 4.4-fold higher B uptake rates and a 5.3-fold B transport rates than 2xCt plants, while the values of these parameters did not exceed 2.0-fold in the 4x+B plants vs. the 4xCt ones (**Figure 2**).

#### Boron Transporters Expression in Roots

**FIGURE 3:**
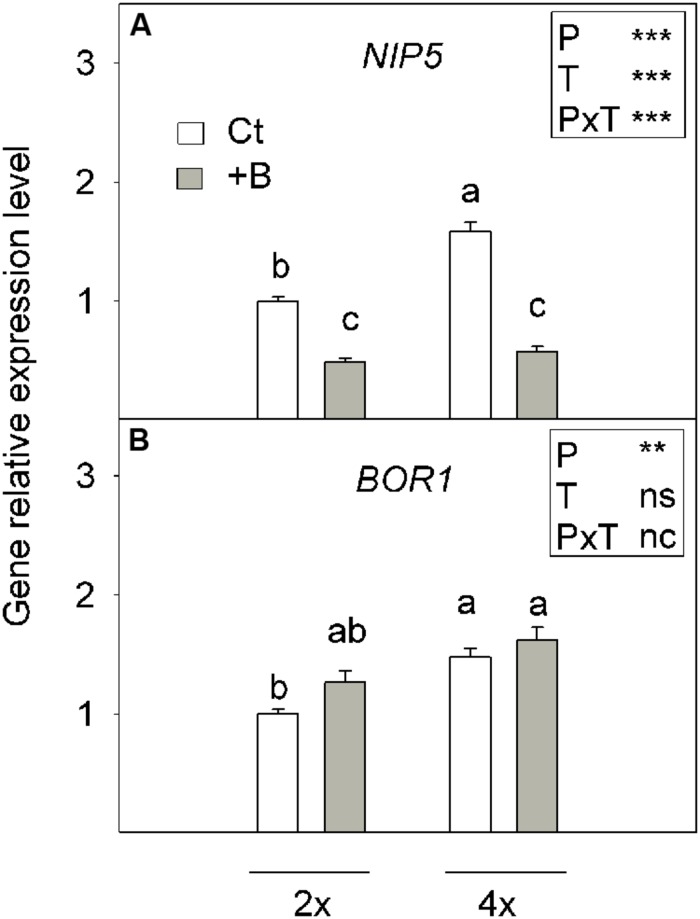
**Relative gene expression level of **(A)***NIP5* and (B) *BOR1* genes measured by qRT-PCR analysis in roots of the 2x and 4x CC seedlings grown *in vitro* (Experiment 1) for 45 days in nutrient media supplemented with 50 μM (Ct) or 400 μM (+B) H_3_BO_3_.** Values are the means ± SE of nine plants (*n* = 9). Ploidy (P) and treatment (T) effects tested by two-way ANOVA are indicated as follows: ^∗∗^*P* < 0.01; ^∗∗∗^*P* < 0.001; ns, not significant; nc, not considered. Different letters indicate significant differences at *P* < 0.05 using Duncan’s multiple range test.

The expression pattern of the genes related in B homeostasis were compared between 2x and 4x roots subjected for 45 days to the control treatment (Ct) or to the high B supply (+B). *NIP5* expresion was higher in 4x roots than in 2x ones under normal conditions (Ct). When subjected to high B supply (+B), the expression level of this gene decreased in both ploidy levels, but the greatest descent was recorded in 4x+B roots (63.3% lower than 4xCt). Nevertheless, the transcript levels reached under +B treatment were similar between 2x and 4x roots (**Figure 3A**).

We also monitored BOR1 expression, which differed between ploidy levels when compared Ct treatments, and was slightly higher in the 4xCt plants than in the 2xCt ones. However, no differences were found between the *BOR1* expression levels in plants grown under B excess (+B) and their corresponding Ct plants (**Figure 3B**).

#### Cellular B Allocation in Soluble and Insoluble Plant Fractions

Plants contain B in both soluble and insoluble forms. The soluble fraction represents B localized in both the free space (apoplast) and the protoplast, while the insoluble fraction corresponds to B bound to cell walls. Control seedlings of both ploidy levels displayed similar B concentrations in the soluble fractions, of either leaves or roots (**Table 4**). B excess (+B) sharply increased the B concentration in the soluble fraction of leaves compared to the Ct ones. Related to Ct, the sharpest B rise in this fraction was recorded for the 2x+B leaves (6.6-fold), whereas was lower in 4x+B leaves (3.5-fold). A similar but less marked effect also occurred in roots (2.1- and 2.8-fold increases for the 2x+B and 4x+B roots, respectively). Finally, the B concentration located in the cell wall of leaves and roots was not significantly affected by either ploidy or treatment (**Table 4**).

**Table 4:** Boron (B) concentration (μg g^-1^ DW) in soluble and cell wall fractions measured in leaves and roots of the 2x and 4x CC seedlings grown *in vitro* (Experiment 1) for 45 days in nutrient solutions supplemented with 50 μM (Ct) or 400 μM (+B) H_3_BO_3_.

	2x		4x		ANOVA		
			
	Ct	+B	Ct	+B	P	T	PxT
**Leaf**							
Soluble	72.3^c^	475.6^a^	77.8^c^	273.0^b^	^∗^	^∗∗∗^	^∗^
Cell wall	36.4	42.1	35.7	43.8	ns	^∗^	nc
**Root**							
Soluble	17.4^b^	36.7^a^	13.4^b^	37.8^a^	ns	^∗∗∗^	nc
Cell wall	13.9	18.3	17.2	19.1	ns	ns	nc


*Values are the means of six plants (*n* = 6). Ploidy (P) and treatment (T) effects tested by two-way ANOVA are indicated as follows: ^∗^*P* < 0.05; ^∗∗∗^*P* < 0.001; ns, not significant; nc, not considered. Different letters in rows indicate significant differences at *P* < 0.05 using Duncan’s multiple range test.*

#### Comparative Histological Study of 2x and 4x Roots

The developmental root pattern in CC is similar to other woody perennial species, where three zones can be distinguished. The white root tip corresponds to the meristematic and elongation zone, where tissue remains undifferentiated and cell walls lack hydrophobic deposits (suberin or lignin). The maturation zone is found above this area, where cell layers undergo differentiation, and suberin and lignin deposits in cell walls start to appear. The following part is the secondary growth zone, where the vascular cylinder is fully differentiated.

The CC seedling roots grown *in vitro* clearly showed these zones, but some differences appeared between the 2x and 4x genotypes. To the unaided eye, the 4x root tips looked shorter and thicker than in the 2x ones, and the transition from the white tip to the mature segment took place within a shorter length to the root tip in the 4x roots than in the 2x ones, with differences observed in structure and tissue development. To test this, root anatomy was examined by comparing segments at different distances from the root apex under the microscope, and specific fluorescent stainings were used to detect lignin or suberin in cell walls. With these dyes, the root epidermis exodermis and the vascular cylinder were efficiently stained, while the root cortex remained dark.

**FIGURE 4:**
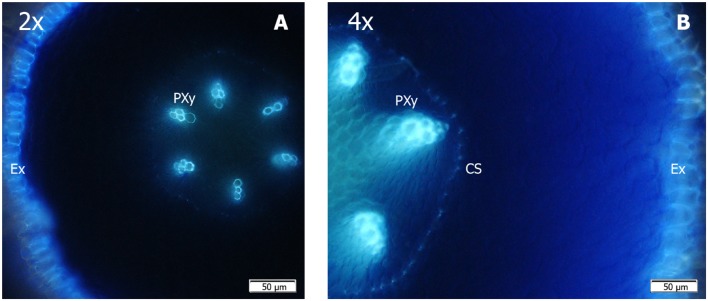
**Free-hand cross-sections of the roots of **(A)** the 2x and **(B)** 4x CC seedlings grown *in vitro*, taken at 9–12 mm from the root apex, cleared in chloral hydrate, stained with berberine hemisulfate, post-stained with aniline blue and visualized under the UV light of a fluorescence microscope.** Casparian strip is clearly differentiated in the 4x root, while is slightly visible in the 2x root. PXy, Protoxylem; CS, Casparian strip; Ex, Exodermis.

At shorter distances to the root tip (<3 mm), we observed no fluorescence in either the 2x or 4x roots, which indicates that both ploidy levels shared at least 3 mm of the meristematic tissue that lacked wall deposits. The earlier identifiable structures were the epidermis and the first protoxylem vessels, which appeared in sections from both genotypes at 3–6 mm from the tip. In the sections taken from 6 to 9 mm from the tip, the epidermis, exodermis, and protoxylem were detected at both ploidy levels, but the developmental stage was more advanced in the 4x root sections than in the 2x ones. At 9–12 mm from the apex, the Casparian strip, located on the endodermis cell layer, was clearly identifiable as dots, which surrounded the vascular cylinder in the 4x roots (**Figure 4B**), while it was only slightly detected in the 2x roots (**Figure 4A**). At a distance of 12–15 mm from the tip, the Casparian strip and the vascular cylinder appeared in an advanced stage of development. Thus the tissue differentiation in the 4x roots was presumed to take place in a shorter distance from the root tip in 4x than in 2x. Moreover, the root tip exodermis was broader in the 4x genotype (**Figure 4B**) than in 2x (**Figure 4A**), and the suberin deposition in the cell walls also appeared thicker in the former.

### Boron Excess Effect on 2x and 4x Plants

#### Previous Description of Plant Morphology

**FIGURE 5:**
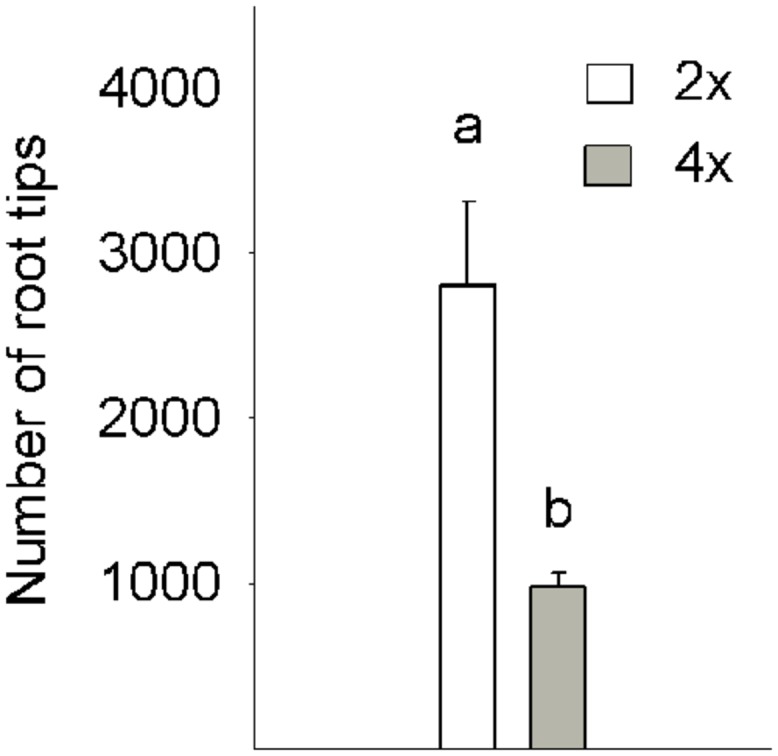
**Number of root tips in 6-month-old 2x and 4x CC seedlings grown in greenhouse conditions.** Values are the means ± SE of three plants (*n* = 3). Different letters indicate significant differences at *P* < 0.05 using Duncan’s multiple range test.

At the beginning of the experiment, the 6-month-old 2x and 4x seedlings had similar DW (**Table 5**) and shoot architecture, since height and leaf number did not differed between them (data not shown). However, the root system morphology was significantly influenced by ploidy. The 4x roots appeared thicker, shorter and had fewer secondary rootlets. Consecuently, the number of root tips in the 2x seedlings was 2.9-fold greater than in the 4x plants (**Figure 5**).

**Table 5:** Dry weight (g) of organs and whole plant of the 2x and 4x CC seedlings grown under greenhouse conditions (Experiment 2) for 10 weeks in nutrient solutions supplemented with 50 μM (Ct) or 800 μM (+B) H_3_BO_3_.

Dry weight	2x		4x		ANOVA		
			
	Ct	+B	Ct	+B	P	T	PxT
Leaf	9.9^a^	8.5^a^	11.8^b^	12.0^b^	^∗^	ns	nc
Stem	28.2^a^	19.0^b^	35.9^a^	34.3^a^	^∗^	^∗^	ns
Root	17.1	15.2	20.2	15.0	ns	ns	nc
FPDW (1)	55.2^a^	42.7^b^	67.9^a^	61.3^a^	^∗^	^∗^	ns
IPDW (2)	20.3	20.3	18.1	18.1	ns	ns	nc
(1) - (2)	34.9^b^	22.4^c^	49.7^a^	43.2^a^	^∗^	^∗^	ns


*The DW of fallen leaves was included in each group of plants. FPDW: DW of the whole plant at the end of treatment; IPDW: DW of the whole plant at the beginning of the treatment; (1–2): difference between the final (FPDW) and initial (IPDW) DW of plants.*

*Values are the means of six plants (*n* = 6). Ploidy (P) and treatment (T) effects tested by two-way ANOVA are indicated as follows: ^∗^*P* < 0.05; ns, not significant; nc, not considered. Different letters in rows indicate significant differences at *P* < 0.05 using Duncan’s multiple range test.*

#### Leaf B Accumulation

**FIGURE 6:**
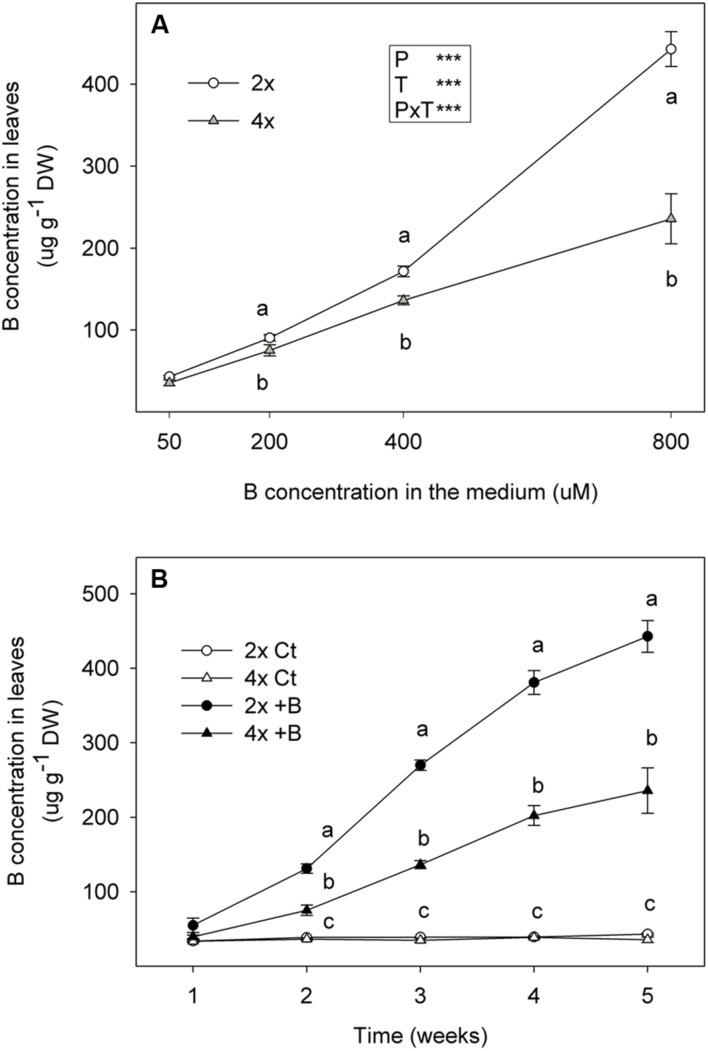
**(A)** Boron concentration (μg g^-1^ DW) in fully expanded leaves from the 6-month-old 2x and 4x CC seedlings grown in greenhouse conditions (Experiment 2) for 5 weeks in nutrient solutions to which 50, 200, 400, or 800 μM H_3_BO_3_ were added. **(B)** Boron concentration (μg g^-1^ DW) in fully expanded leaves from 6-month-old 2x and 4x CC seedlings grown in glasshouse for 5 weeks in nutrient solutions to which 50 μM (Ct) or 800 μM (+B) H_3_BO_3_ were added. Values are the means ± SE of six plants (*n* = 6). Ploidy (P) and treatment (T) effects tested by two-way ANOVA are indicated as follows ^∗∗∗^*P* < 0.001. Different letters indicate significant differences at *P* < 0.05 using Duncan’s multiple range test.

The total B concentration was measured in leaves of the 2x and 4x seedlings grown for 5 weeks under different B supply in nutrient solution. As expected, the B concentration in leaves rose when the H_3_BO_3_ treatment was increased from 50 to 800 μM (**Figure 6A**). However, the 2x leaves accumulated more B than the 4x ones. After 5 weeks, the B concentration in the leaves of 2x plants irrigated with 800 μM H_3_BO_3_ (+B), was 10.3-fold higher than in the 2x plants irrigated with the normal B supply (Ct), while this value was 6.6-fold higher in the leaves of 4x+B plants than in the 4xCt ones. Thus, the B content in the 4x+B leaves was 46.8% lower than in the 2x+B ones (**Figure 6B**).

#### Effects of B Excess on Growth, Leaf Damage, and Abscission

Seedlings irrigated with 800 and 50 μM H_3_BO_3_ (+B and Ct, respectively) were maintained up to 10 weeks to better check the effects of these treatments on growth, leaf toxicity damage and leaf abscission. As shown in **Table 5**, treatment +B did not affect the plant DW of the 4x plants when compared to the corresponding control (4xCt), while it lowered the DW of the 2x plants in comparison to 2xCt. Considering the growth of plants as the increase in DW during the experimental period (measured by the difference between the DW of plants at the end and the beginning of treatment), no differences were found between 4xCt and 4x+B seedlings, whereas 2x+B seedlings had a 36% lower biomass than 2xCt. Therefore, the B excess conditions reduced the growth of the 2x plants, while did not affect the 4x ones.

**FIGURE 7:**
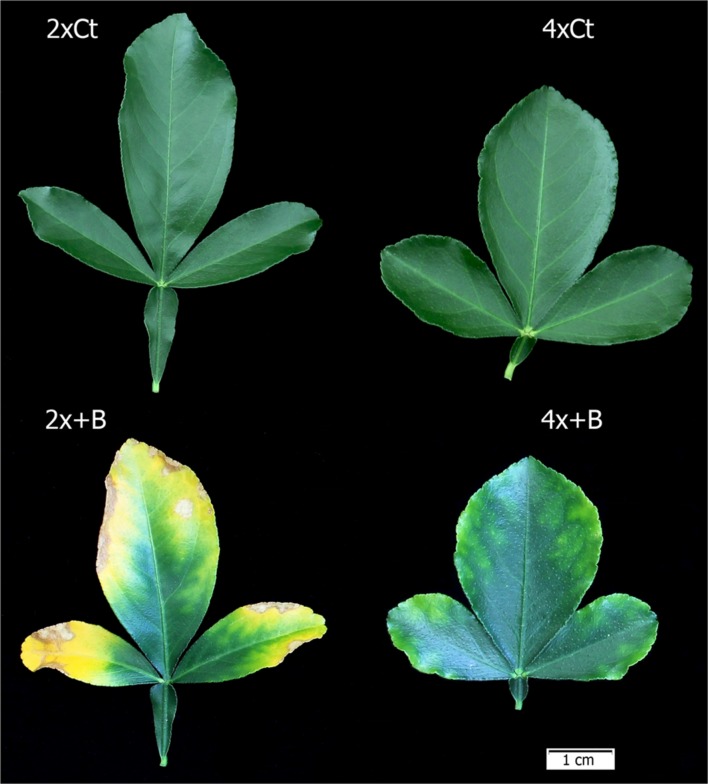
**Visual symptoms of B toxicity in leaves from the 6-month-old 2x and 4x CC seedlings grown in greenhouse conditions (Experiment 2) for 10 weeks in nutrient solutions added with 50 μM (Ct) and 800 μM H_3_BO_3_ (+B)**.

**FIGURE 8:**
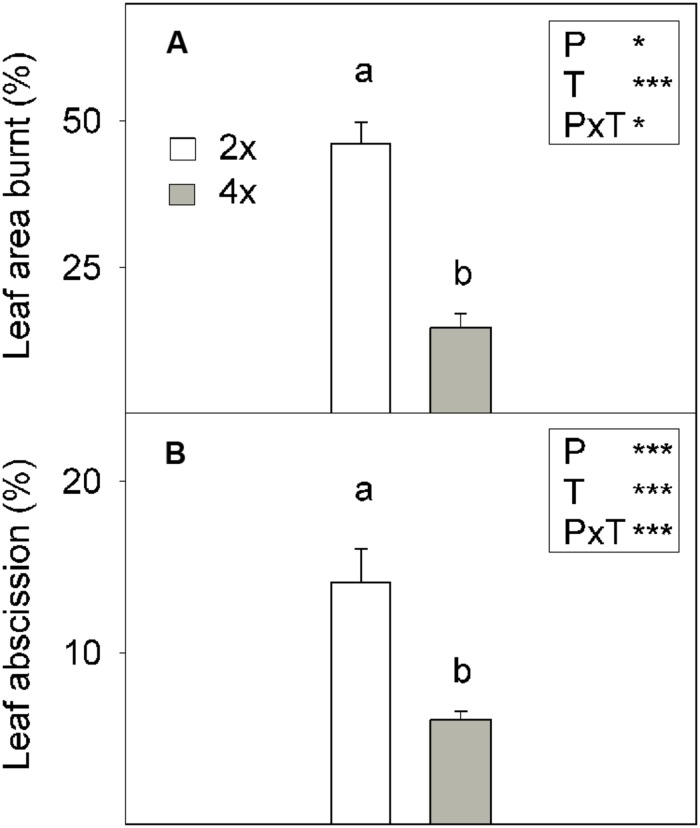
**(A)** Leaf damage (%) and **(B)** leaf abscission (%) in the 6-month-old 2x and 4x CC seedlings grown in greenhouse conditions (Experiment 2) for 10 weeks in nutrient solutions to which 800 μM (+B) H_3_BO_3_ was added. Values are the means ± SE of six plants (*n* = 6). Ploidy (P) and treatment (T) effects tested by two-way ANOVA are indicated as follows: ^∗^*P* < 0.05; ^∗∗∗^*P* < 0.001. Different letters indicate significant differences at *P* < 0.05 using Duncan’s multiple range test.

The 2x+B plants showed much more severe B toxicity symptoms in their leaves than the 4x+B plants (**Figure 7**), as confirmed by the percentages of damaged leaf area (**Figure 8A**). Thus in the 2x+B plants, the burnt leaf area was 3.1-fold greater than in 4x+B. High B supply (+B) also induced leaf abscission in both genotypes, although this effect was more marked in the 2x plants, which showed a 2.2-fold greater abscission percentage than the 4x ones (**Figure 8B**).

### Boron Excess in Field Conditions: Effect of Rootstock Ploidy on B Accumulation in the Grafted Variety

**FIGURE 9:**
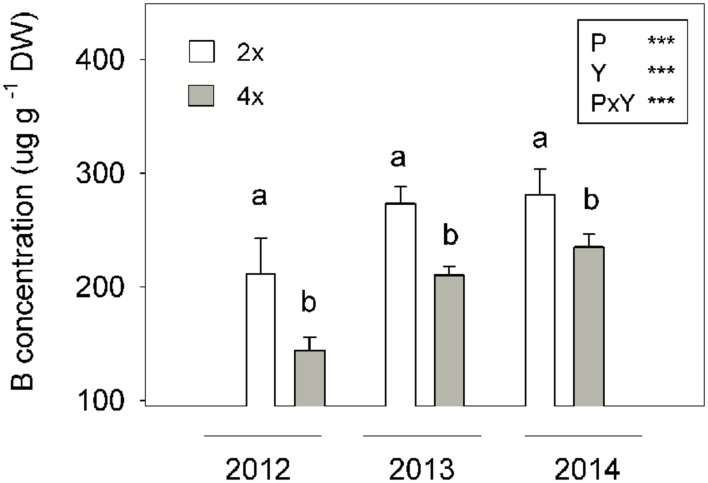
**Boron (B) concentration (μg g^-1^ DW) in 9-month-old spring flush leaves of 5-year-old VL orange trees grafted onto the 2x and 4x CC rootstocks grown under field conditions (Experiment 3).** Ploidy (P) and year (Y) effects tested by two-way ANOVA are indicated as ^∗∗∗^*P* < 0.001. Values are the means ± SE of six leaf samples pooled from the four trees of each block. Different letters indicate significant differences at *P* < 0.05 using Duncan’s multiple range test.

The B concentration, measured over three seasons in 9-month-old spring leaves of VL orange trees, grown under field conditions, was significantly affected by rootstock ploidy (**Figure 9**). In 2012, the leaves of VL grafted onto 2x CC rootstocks (VL/2xCC) presented a 1.5-fold higher B concentration than the leaves of the VL grafted onto 4x CC rootstocks (VL/4xCC). This trend persisted in 2013 and 2014 (1.3- and 1.2-fold, respectively), even when the B concentration tended to increase in trees over time (**Figure 9**). However, in this experiment, no B-toxicity symptoms were observed in either VL/2xCC or VL/4xCC. Tree height, canopy volume and production reached in 2015 were 10.8, 42.2, and 53.3%, respectively, lower in the VL/4xCC trees than in the VL/2xCC ones, while yield efficiency did not differ between them (**Table 6**).

**Table 6:** Characteristics of 6-year old VL orange trees grafted onto the 2x and 4x CC rootstocks grown under field conditions (Experiment 3).

	2x	4x	ANOVA
Tree heigth (m)	1.8^a^	1.6^b^	^∗^
Canopy volume (m^3^)	3.1^a^	1.8^b^	^∗∗^
Production (kg)	15.4^a^	7.2^b^	^∗∗∗^
Eficiency (kg m^-3^)	5.0	4.1	ns


*Values are means of 12 plants (*n* = 12). Significant differences for ploidy (P) tested by ANOVA are indicated as follows: ^∗^*P* < 0.05; ^∗∗^*P* < 0.01; ^∗∗∗^*P* < 0.001; ns, not significant. Different letters in rows indicate significant differences at *P* < 0.05 using Duncan’s multiple range test.*

## Discussion

In the first experiment, the plants treated with high B increased B concentrations in all plant organs, however, B was preferentially accumulated in leaves, while relatively low concentrations were found in stems and roots (**Figure 1**). These data are in accordance with those previously reported (Papadakis et al., 2004) and indicate that a high rate of absorbed B is transported from roots to leaves, driven by the transpiration stream. Further B remobilisation from leaves to other organs via the phloem is limited. Therefore, this element is scarcely redistributed in plants (Brown and Shelp, 1997). Boron immobility in leaves has been considered a defense mechanism of plants against B toxicity, since vegetative growth may continue in young plant organs, while excess B accumulates in old leaves (Nable et al., 1997). As the phloem is unable to transport B, this element is retained in leaf margins from mature leaves, which develop burns under excess B conditions. Notwithstanding, leaves were still able to maintain enough healthy photosynthetic leaf area (Nable et al., 1997). Interestingly, despite the lack of plant growth affection by B supply in both genotypes (**Table 2**), 2x+B seedlings registered higher leaf B concentration than 4x+B ones (**Figure 1**), which is in agreement with previous observation reported by Papadakis et al. (2004). Consequently under B excess conditions, B uptake and root-to-shoot transport rates were higher in the 2x seedlings than in the 4x ones.

Initially, there could be three possible explanations for these differences:

(a) Regulation of membrane transport proteins for B uptake and root-to-shoot transport: acquisition of B by root cells is associated with the activity of some aquaporin family members, especially CiNIP5, which has been characterized in CC (An et al., 2012). This gene codifies a BA channel, which facilitates B uptake under limited B supply by increasing its expression level in B-deficient roots. In contrast, this gene is down-regulated in +B seedlings, which agrees with a previous report by Martinez-Cuenca et al. (2015) on *C. macrophylla*. Our results suggest that, at increased external B levels, citrus roots diminished B uptake efficiency, irrespectively, of ploidy.
The expression level of *BOR1*, which modulates B xylem loading, and therefore B translocation from roots to shoots (Takano et al., 2002), did not differ between Ct and +B roots of both genotypes or between 2x+B and 4x+B roots. In citrus, functional *CmBOR1* characterisation has been recently achieved in *C. macrophylla* by Cañon et al. (2013). *CmBOR1* expression in roots is not affected by high external B concentrations, and the activity of this gene is responsible for the good capacity of citrus to accumulate B in leaves, even under B excess conditions (Cañon et al., 2013; Martinez-Cuenca et al., 2015). Thus in plants that receive a high B supply, ploidy does not affect the system for transporting B from roots to leaves.
In contrast, some reports suggested that the homologous genes of the above-mentioned are involved in tolerance mechanisms to B toxicity in some plants. Schnurbusch et al. (2010) showed a relation between *HvNIP2;1* gene expression and B toxicity tolerance in barley, while Tanaka et al. (2011) suggested the importance of AtNIP5;1 degradation for plant acclimation to high B conditions. Moreover, Sutton et al. (2007) identified the *BOR1* ortholog *Bot1* as responsible for Sahara barley’s B-toxicity tolerance, since its amplification facilitated B efflux from roots to maintain less shoot B accumulation. Reid (2007) reported that B-tolerant barley and wheat cultivars are able to maintain low B concentracions in shoots and roots by the active efflux mediated by HvBOR2 and TaBOR2. However, the present data indicate that at high B levels in culture medium, the expression of *CiNIP5* and *BOR1* was similar in 2x and 4x seedlings (**Figure 3**), thus the lower leaf B concentration in the 4x plants would be unlikely due to differences in B carrier-mediated transport.
(b) Cellular allocation: anatomic differences between the organs of plants 2x and 4x (Allario et al., 2011) support the possibility that B excess tolerance is related to the amount of B bound to cell walls, which would lower the level of mobile B that is freely available inside leaf tissues. These forms correspond to insoluble and soluble B fractions, respectively. Most of the soluble fraction is constituted by B extracted in water, which is mainly localized in the plant free space or apoplast, while the remainder soluble B, extracted with organic solvents, belongs to the B located inside the protoplast, and is linked to organic molecules (Liu et al., 2013). Soluble B is the major fraction of B in the +B seedling organs of both genotypes, and appears to increase according to the total B absorbed by the plant. Finally, the insoluble fraction represents cell wall-bounded B, linked to pectic polysaccharides (Liu et al., 2013), and its concentration in either roots or leaves was similar in both genotypes, irrespectively, of treatment. This suggests a critical concentration of insoluble B in which the saturation of binding sites was accomplished while the soluble fraction was readily translocated from roots to leaves, where it accumulated under B excess conditions to cause visible leaf damage. The ability of cell walls to retain B enables seedlings to block a large part of this element in an insoluble form, thus preventing its entry in the cytoplasm, and therefore protecting cells from B toxicity. Although the insolubilisation of B outside the cytoplasm has been proposed as a general mechanism of B excess tolerance (Martinez-Cuenca et al., 2015), the B amounts linked herein to cell walls by the weight unit of either roots or leaves were similar in seedlings 2x and 4x. Consequently, B insolubilisation does not seem to be a determinant of the differences noted between both genotypes in soluble leaf B content.
(c) Morphology and anatomy of roots: finally, we checked whether the differences in these traits between the roots of 2x and 4x genotypes could affect B uptake and transport. The 4x roots, which have a lower SRL and longer average root diameters, exhibited a relatively thicker exodermis than the 2x roots (**Figure 4**). Exodermis development is linked to enhanced deposition of suberin layers, which is more marked in the 4x roots than in the 2x ones. This agrees with a previous report which indicated that citrus rootstock genotypes with short average root diameters and high SRL tend to display less exodermal wall thickening (Eissenstat and Achor, 1999). Secondary wall development of the exodermis can further enhance resistance to water and ion flow by blocking plasmodesmata, thereby limiting symplasmic connections of the plasma membrane of exodermal cells (Walker et al., 1984). Therefore under these conditions, the major sites of ion uptake in the symplasm are the thin-walled passage cells of the exodermis (Walker et al., 1984). It has been reported that *Lp*_r_ is inversely related to root cortical thickness and to the degree of suberisation of the exodermis (Huang and Eissenstat, 2000). In this way, the exodermis and cortical tissue of the 4x genotype can impose greater resistance to water and solute movement on the radial pathway than in the 2x genotype. These features might partly explain the differences observed in BA uptake between 2x and 4x roots.

Most water and nutrient uptake is generally attributed to actively growing root tips, even though suberised and lignified portions can constitute the vast majority of the root surface area for woody species (Gambetta et al., 2013). Therefore, differences in morfology and suberization pattern between root tips of both genotypes could influence BA uptake and transport. It has been commonly assumed that most BA travels apoplastically across the root cortex to reach the Casparian strip, which is then overcome through the cytoplasm of endodermal cells. However, the transport capacity (including influx and efflux) of BA across the plasmalemma of these cells is not likely to be sufficient for xylem loading with the amount of BA that plants require. The Casparian strip, formed within the transverse and radial walls of endodermal cells, in which suberin and lignin are deposited, is present throughout the root, except for a few millimeters right after the root tip. Over these, the band develops and approximately coincides with protoxylem maturation (Peterson and Lefcourt, 1990). Thus at a variable distance from roots, the apex layers of suberin are deposited on the inner surface of the walls of endodermal cells, which creates a lamellar structure that isolates their protoplasts from the apoplast. Presence of suberin lamellae is thought to prevent endodermal cells from uptaking solutes from the apoplast. Consequently, a high proportion of total apoplastic solute flux might enter by the root tip, where the Casparian strip has not yet developed (Enstone and Peterson, 1992). This pathway has been proposed to be a preferred option for Ca^2+^ movement to the xylem, whereby roots can fulfill the calcium demand of shoots without affecting the intracellular Ca^2+^ concentration (White, 2001). It is quite likely that a similar process occurs with BA since its behavior in the plant resembles some aspects of that of Ca^2+^. Nevertheless, a recent mathematical modeling of B uptake in Arabidopsis specifies that the main B entrance to the root is not the very tip, but a more mature region (Shimotohno et al., 2015). Arabidopsis root anatomy differs in some important anatomical features from woody species (Nawrath et al., 2013) although, root suberization in this specie is also inversely proportional to water uptake (Ranathunge and Schreiber, 2011), and modifies mineral nutrition (Baxter et al., 2009). Thus our proposal might be somewhat operative in this specie. Enhanced salinity tolerance leaded by altered Na^+^ and K^+^ uptake in 4x Arabidopsis are attributed to root modification induced by genome duplication (Chao et al., 2013), but related changes in suberization pattern remain unexplored.

Moreover, Casparian strip formation in 4xCC roots seems to start earlier than in 2xCC roots (**Figure 4**), consequently the incipient deposition of lignin and suberin in 4x appeared at a shorter distance from the apex than in 2x. In agreement to our proposal, Barrios-masias et al. (2015) point out that in grapevine rootstocks (*Vitis* spp.) with shorter roots, suberin is formed closer to the root tip, and root tissue maturation is earlier, reducing water uptake and tree vigor in response to the resulting decrease in root hydraulic conductivity. Root suberization is also related to excess B response in soybean, suggesting that might be a key mechanism operating to reduce water and B transport (Ghanati et al., 2005). This characteristics shown by the 4x genotype, could determine its low capacity to absorb and transport water from roots to leaves.

Experiment 2 confirms the greater tolerance of 4x plants vs. 2x since the latter accumulated 1.9-fold more B in leaves than the former (**Figure 6B**), and suffered a biomass reduction, which did not occur in the 4x plants (**Table 5**). Thus, the greenhouse-tested 2x plants showed different growth behavior when faced with excess B from the 2x seedlings cultured *in vitro* (Experiment 1), in which DW was not significantly affected by excess B. This apparent contradiction can be explained by the difference in exposure time to +B treatment, which was longer in Experiment 2 (10 weeks) than in Experiment 1 (45 days). Moreover, the BA concentration used in the nutritive solution of +B treatments in the greenhouse assay was higher (800 μM) than the *in vitro* one (400 μM).

The greater sensitivity of the 2x genotype to excess B compared to 4x was also evidenced by leaf damage and abscission, which were more marked in the 2x plants as a result of +B treatment (**Figure 8**).

The above differences found between 2x and 4x genotypes in response to excess B could be partly due to a characteristic of the root system of 2x plants, which had 2.9-fold more tips than 4x plants, and therefore more points for BA absorption (**Figure 5**). It has been proposed that some ions may reach the xylem through the apoplast as lateral roots emerge since, during their formation, endodermal cells divide and new walls lack of Casparian strip (Peterson and Lefcourt, 1990; White, 2001). However, this effect is only temporary, while Casparian strip are deposited in incipient lateral roots, and rapidly become continuous with those of parent roots (White, 2001). Consequently, as occurs with Ca^2+^ ions, the BA transport by the apoplastic flux at sites may be significant where lateral roots penetrate the endodermis.

Finally, in the field experiment, where VL orange trees, grafted onto either rootstocks 2x or 4x CC, were irrigated with water with a high B level, the B concentration in leaves was monitored for 3 years to confirm outdoors the ability of the 4x roots to exclude B from leaves. As expected, the the VL/4xCC trees had lower B concentrations in leaves than those grafted onto the 2x rootstock, for all the sampled years (**Figure 9**), suggesting that 4xCC is a candidate rootstock to relieve citrus trees from B toxicity. The VL/4xCC trees presented a smaller canopy and lower yields, but similar yield efficiency, compared with VL/2xCC (**Table 6**). Obviously, these traits are not dependent on the B levels in leaves, but are rather the consequence of the differences between roots 2x and 4x CC, as in *Lp*_r_, which affects tree growth and yield (Syvertsen and Graham, 1985). Investigations performed in 2x and 4x Rangpur lime (*C. limonia* Osb.) grafted with 2x orange showed that 4x conferred enhanced tolerance to drought mediated by specific genes, including the *CsNCED1* gene, which led to increased root-to-shoot transfer of ABA (Allario et al., 2013). In Arabidopsis, the inhibition of root growth caused by B-toxicity is mediated by ABA signaling, cell wall modifications and the inhibition of genes encoding water transporters, concluding that B-toxicity triggered a water-stress response associated with root growth inhibition (Aquea et al., 2012). Consequently, it can not be excluded that higher ABA biosynthesis in 4x roots may be also involved in a more efficient regulation of gas exchange of scions grown on 4x root genotypes, contributing to enhance B tolerance.

## Conclusion

Tetraploid plants present a lower capacity of uptaking and transporting B than 2x plants, which results in a reduced B concentration in the leaves of the former than in those of the latter. This effect seems to be the result of the differences in the morphological and histological traits of roots.

## Author Contributions

Conceived and designed the experiments: MR, EP, MM. Performed the experiments: MR. Contributed reagents/materials/analysis tools: AQ, BM, PA. Analyzed the data: MR, EP, MM. Wrote the paper: MR, EP, MM. Reviewed the manuscript: RM, LN.

## Conflict of Interest Statement

The authors declare that the research was conducted in the absence of any commercial or financial relationships that could be construed as a potential conflict of interest.

## Abbreviations

2x: diploid
4x: tetraploid
*A*co_2_: net CO_2_ assimilation rate
BA: boric acid
B: boron
BOR: boron transporter
CC: Carrizo citrange
DW: dry weight
E: transpiration rate
FW: fresh weight
*Lp*_r_: radial hydraulic conductivity
NIP: nodulin 26-like intrinsic protein
qRT-PCR: quantitative reverse transcription polimerase chain reaction
SRL: specific root length
VL: Valencia late
